# Superoxide dismutase reduces monosodium glutamate-induced injury in an organotypic whole hemisphere brain slice model of excitotoxicity

**DOI:** 10.1186/s13036-020-0226-8

**Published:** 2020-02-04

**Authors:** Rick Liao, Thomas R. Wood, Elizabeth Nance

**Affiliations:** 1 Department of Chemical Engineering, University of Washington, 3781 Okanogan Lane NE, Seattle, WA 98195 USA; 2 Department of Pediatrics, University of Washington, Seattle, WA USA; 3 Department of Radiology, University of Washington, Seattle, WA USA; 4 Center on Human Development and Disability, University of Washington, Seattle, WA USA

**Keywords:** Oxidative stress, Peroxynitrite, Mitochondria, Neuroinflammation, Hyperosmolar stress, 8-hydroxy-2-deoxyguanosine, Antioxidant, Ex vivo

## Abstract

**Background:**

Knowledge of glutamate excitotoxicity has increased substantially over the past few decades, with multiple proposed pathways involved in inflicting damage. We sought to develop a monosodium glutamate (MSG) exposed ex vivo organotypic whole hemisphere (OWH) brain slice model of excitotoxicity to study excitotoxic processes and screen the efficacy of superoxide dismutase (SOD).

**Results:**

The OWH model is a reproducible platform with high cell viability and retained cellular morphology. OWH slices exposed to MSG induced significant cytotoxicity and downregulation of neuronal excitation-related gene expression. The OWH brain slice model has enabled us to isolate and study components of excitotoxicity, distinguishing the effects of glutamate excitation, hyperosmolar stress, and inflammation. We find that extracellularly administered SOD is significantly protective in inhibiting cell death and restoring healthy mitochondrial morphology. SOD efficacy suggests that superoxide scavenging is a promising therapeutic strategy in excitotoxic injury.

**Conclusions:**

Using OWH brain slice models, we can obtain a better understanding of the pathological mechanisms of excitotoxic injury, and more rapidly screen potential therapeutics.

## Introduction

Glutamate excitotoxicity is a common hallmark in many neurological diseases, including stroke, traumatic brain injury (TBI), and depression [1–3]. In excitotoxicity, excessive glutamate release over-activates neuronal postsynaptic glutamatergic N-methyl-D-aspartic acid (NMDA) receptors, causing sodium and calcium to flood into the neuron, generation of reactive oxygen species (ROS), and mitochondrial damage, ultimately initiating neuronal death processes [4–6]. Excitotoxicity can mediate cell death through both acute necrosis due to cell swelling upon uptake of sodium and chloride, and apoptosis involving calcium-induced downstream pathways [7, 8]. Combating excitotoxic cell death holds potential in ameliorating neuronal death in many neurological diseases.

Enzymes in their native form are actively studied for their role in managing neurological damage, specifically involving oxidative stress [9–12]. Exogenously delivered antioxidant enzymes can help reestablish redox equilibrium within cells to mitigate excitotoxic brain damage. Catalase, superoxide dismutase (SOD), glutathione peroxidase, and other peroxiredoxins all work to eliminate oxidative agents including hydrogen peroxide (H_2_O_2_), superoxide anion radical (O_2_^−^), and peroxynitrite anion (ONOO^−^) [13]. SOD, which converts O_2_^−^ into H_2_O_2_ and oxygen, has been widely studied and shows therapeutic potential in multiple disease models that exhibit excitotoxicity, including in vitro NMDA-induced neuronal cell culture, and in vivo middle cerebral artery occlusion models in rats [14–17].

In this study, we use ex vivo organotypic whole hemisphere (OWH) brain slices as a high-throughput tool for monosodium glutamate (MSG)-induced excitotoxicity disease model development and therapeutic efficacy screening of SOD. OWH brain slice models serve as an intermediate alternative to neuronal/glial cell cultures that fail to capture the 3D and cell-type complexity of the brain microenvironment, and in vivo animal models that suffer from confounding factors that limit mechanistic, systematic analysis [18].

## Materials and methods

### Preparation for brain slice culturing

All experiments were approved by the University of Washington Institutional Animal Care and Use Committee, and adhere to the guidelines of the NIH Guide for the Care and Use of Laboratory Animals [19]. On postnatal (P) day 14, healthy Sprague Dawley (SD, *Rattus norvegicus*) rats were injected with 100 μL pentobarbital, followed by rapid decapitation with surgical scissors once the body was non-responsive. After removing the brain under sterile conditions, the brain was split into hemispheres with a sterile razor blade and sliced into 300 μm sections with a McIlwain tissue chopper (Ted Pella). Brain slices were separated in dissecting media (0.64% w/v glucose, 100% HBSS (Hank’s Balanced Salt Solution), 1% penicillin-streptomycin). Brain slices containing the hippocampus were transferred onto 35-mm 0.4-μm-pore-sized membrane inserts (Millipore Sigma), and placed within a 6-well plate (CytoOne) containing 1 mL 37 °C pre-heated slice culture media (SCM; 50% MEM (minimum essential media), 50% HBSS, 1% GlutaMAX, and 1% penicillin-streptomycin). For hippocampal slice culture experiments, only the hippocampal sections from 6 adjacent slices were transferred to the membrane insert to obtain approximately the same amount of organotypic tissue as a single whole hemisphere slice. All media added to slices was pre-warmed at 37 °C. MEM was purchased from Life Technologies, glucose from Sigma, and HBSS, GlutaMAX, and penicillin-streptomycin from Gibco. The slices rested overnight in a CO_2_ incubator (ThermoFisher Scientific) at 37 °C with constant humidity, 95% air, and 5% CO_2_ to equilibrate after the mechanical stress of slicing before continuing experiments.

### Sample preparation for lactate dehydrogenase (LDH) cytotoxicity

After slices rested overnight, supernatant was collected (time t = − 3 h) and replaced with SCM containing 1–1000 mM MSG (L-glutamic acid monosodium salt hydrate, Sigma), 1000 mM NaCl (sodium chloride, Sigma), or 100 ng/mL LPS (lipopolysaccharide O111:B4, Sigma) for disease induction, if applicable. SCM without any additional exposures served as the non-treated (NT) control, and SCM containing 1% Triton-X-100 (TX, Cayman Chemical), a surfactant that induces death and membrane permeabilization of all cells, served as the maximum death control. For slice culture studies, the end of the 3 h incubation was defined as time t = 0 h. At t = 0 h, the exposure-containing SCM was collected and replaced with normal SCM. At the 0 h or later specified timepoint, 100 μL containing 0.01 mg or 0.1 mg of SOD1 (copper/zinc SOD from bovine erythrocytes, Sigma) suspended in SCM, or pure SCM as a control, was gently added to the top of the brain slice using a cut-tip pipet. NT control slices were incubated with NT SCM throughout the 3 h incubation and 24 h culturing. TX control slices were incubated with NT SCM during the 3 h incubation, and then throughout 24 h with TX SCM afterwards. Supernatant collection and media replacement were repeated at time 1 h, 2 h, 4 h, 8 h, and 24 h. All supernatant samples were immediately stored in − 80 °C. For OWH samples, *n* = 6 slices were processed for 1000 mM NaCl and 100 ng/mL LPS, while *n* = 18 slices were processed for all other conditions. For OHC samples, *n* = 3 slices were processed. For conditions with *n* = 18 slices, *n* = 9 slices were from male rats, and *n* = 9 slices were from female rats. All other slice experiments in this manuscript were from female rats.

Supernatant samples were removed and thawed at room temperature (RT) to conduct lactate dehydrogenase (LDH) assays (Cayman Chemical). LDH is a cytosolic enzyme that is released from the degrading membrane of dying or dead cells. Following the manufacturer’s instructions, 100 μL of sample supernatant was added to 100 μL of LDH reaction buffer in technical triplicates to 96-well plates on ice, and the plates were transferred to a stir plate in a 37 °C incubator. After 30 min, the plates were placed on ice, and absorbance was measured at 490 nm (A_490_) on a SpectraMax M5 UV-Vis Spectrophotometer (Molecular Devices) to detect the production of colorimetric formazan. Percent cytotoxicity was calculated using Eq. 1.

1 $$ \% cytotoxicity=\frac{cumulative\  LDH\  abs\  of\ sample}{24h\  cumulative\  LDH\  abs\  of\ reference}\times 100\% $$

### OWH sample preparation for immunofluorescence

Slice culture preparation for immunofluorescence (IF) was similar to LDH slice preparation, except without supernatant replacement after t = 0 h. At t = 6 h, brain slices from P14 female rats were fixed in 10% formalin (10% phosphate buffered formalin, ThermoFisher) with 1 mL below membrane insert and 500 μL directly on the slice at room temperature for 1 h. Slices were washed twice with PBS (phosphate buffered saline, Gibco) and stored in 1 mL PBS until IF staining. Recombinant antibodies for neurons (rabbit anti-NeuN 488, Abcam) were prepared 1:250 in PBS with 0.01% Triton-X (PBS+). Primary antibodies for microglia (Wako rabbit anti-Iba1+, Abcam) were prepared 1:250 in PBS containing 0.01% Triton-X (Sigma) and normal goat serum (Sigma). Two hundred fifty microliters primary antibody solutions were added to each tissue section for 6 h at room temperature. Sections were washed twice in PBS. Secondary antibodies for Iba1+ microglia (AF-488 IgG goat anti-rabbit, Invitrogen) were prepared 1:500 in PBS+. For microglia staining, 250 μL secondary antibody solutions were added to each tissue section for 2 h and washed twice in PBS. Sections were stained with 1 mL of 5 ng/mL DAPI (4′,6-diamidino-2-phenylindole, Invitrogen) in PBS, washed twice in PBS, and then stored in PBS at 4 °C until imaged on a Nikon confocal microscope. For confocal imaging, 20x confocal z-stack images set to max intensity projection were obtained for NeuN neuronal and Iba1+ microglial imaging.

### OWH sample preparation for RT-PCR analysis

Slice culture preparation for real time polymerase chain reaction (RT-PCR) was similar to LDH slice preparation. All studies were performed with P14 female rats. Three slices were plated per membrane insert to obtain sufficient tissue for RNA extraction (~ 30 mg) and incubated with exposure-containing SCM (100 mM MSG, 100 mM NaCl, 100 ng/mL LPS, 100 μM NMDA; Supplementary 1000 mM MSG, 1000 mM NaCl) for 3 h. At t = 0 h, supernatant was replaced with 1 mL normal SCM, and 300 μL SCM containing 0.3 mg SOD was added on top of the 3 slices for SOD-treated samples. OWH slices were removed from culturing at t = 6 h. Slices were gently separated from the membrane inserts with a flat spatula and transferred to a 20 mL scintillation vial containing 1 mL of RNALater (Invitrogen) and stored at − 80 °C. After the slices thawed, RNALater was removed and 1 mL of TRIzol was added. RNA extraction was performed following the TRIzol (Invitrogen) manufacturer protocol. Slices were homogenized by pipetting repeatedly with a Pasteur pipet. Chloroform (Sigma) was then added to the tube followed by centrifugation. The top aqueous phase was collected into another tube and underwent a series of centrifugation and washing steps with isopropanol and ethanol. After measuring RNA concentration with Nanodrop, 2 μg RNA was converted to cDNA with the High Capacity RNA to cDNA kit (Applied Biosystems). mRNA expression fold-changes were measured with the SYBR Green RT-PCR kit (Applied Biosystems) for the mRNAs IL-1β (interleukin-1 beta), IL-6, TNF-α (tumor necrosis factor-alpha), Dlg4 (discs large MAGUK scaffold protein 4), EGR1 (early growth response 1), nNOS (neuronal nitric oxide synthase), HMOX1 (heme oxygenase 1), GCLM (glutamate-cysteine ligase modifier subunit), and SOD1 (copper/zinc superoxide dismutase). ΔΔCt values were calculated, with reference to the NT slice control sample with GAPDH (glyceraldehyde-3-phosphate dehydrogenase) as the housekeeping gene. RT-PCR was conducted using technical duplicates or triplicates, and sample size varied from *n* = 3 to *n* = 12 for the datasets presented in the study. Forward and reverse RNA primers (Table 1) were obtained from Integrated DNA Technologies.

**Table 1:** Forward and reverse primer sequences for RT-PCR

Gene	Accession number	Forward primer	Reverse primer
GAPDH	NM_017008.4	GTC GGT GTG AAC GGA TTT	TGT AGT TGA GGT CAA TGA AGG
IL-1β	NM_031512.2	TTC GAC AGT GAG GAG AAT G	GAT GCT GCT GTG AGA TTT G
IL-6	NM_012589.2	GGA GAC TTC ACA GAG GAT AC	GCC ATT GCA CAA CTC TTT
TNF-α	NM_012675.3	CCT CAG CCT CTT CTC ATT C	GGA ACT TCT CCT CCT TGT T
Dlg4	NM_019621.1	CGG GAA CAG CTC ATG AAT A	TCC TTG GTC TTG TCG TAA TC
EGR1	NM_012551.2	CTG ACC ACA GAG TCC TTT	GGT AGT TTG GCT GGG ATA A
nNOS	NM_052799.1	AGC GTC TCC TCC TAT TCT	ACT GAG AAC CTC ACA TTG G
HMOX1	NM_012580.2	CAC ATC CGT GCA GAG AAT	GGC CAT CAC CAG CTT AAA
GCLM	NM_017305.2	CAG TGG GCA CAG GTA AA	GTG AGT CAG TAG CTG TAT GT
SOD1	NM_017050.1	GTG GTG TCA GGA CAG ATT AC	TGG TAC AGC CTT GTG TAT TG

Primers were designed to have an amplicon size between 50 and 150 base pairs, 40–60% GC content, no four consecutive nucleotide repeats, melting temperature (T_m_) between 58 and 60 °C, and no more than three G or C bases among the last 5 nucleotides of the 3′ end.

### OWH sample preparation for mitochondrial morphology

Slice culture preparation for mitochondrial imaging was similar to LDH slice preparation. At t = 0 h, live brain slices from P14 female rats were stained with MitoTracker Deep Red FM (ThermoFisher). Starting with 1 mM MitoTracker stock solution in DMSO, MitoTracker was diluted to a working solution of 500 nM in SCM. After warming to 37 °C, 1 mL SCM with MitoTracker was added below the membrane insert and 100 μL applied on top of each brain slice followed by a 1 h incubation at 37 °C with constant humidity, 95% air, and 5% CO_2_. For SOD-treated slices, the 100 μL SCM with MitoTracker contained 0.1 mg SOD. Slices were then washed once with warmed SCM, and then fixed in 4% formaldehyde (1 mL below membrane insert, 500 μL directly on the slice) at room temperature for 1 h. Slices were washed twice with PBS, stained with 1 mL of 5 μg/mL DAPI in PBS, washed twice in PBS, and then stored in PBS at 4 °C until imaging on a Nikon confocal microscope. For confocal imaging, a 20x confocal scan of each slice was obtained, followed by additional images at 240x (60x lens with 4x zoom) for representative mitochondrial morphology.

### 8-hydroxy-2-deoxyguanosine (8-OHdG) ELISA

After removal of the aqueous phase containing RNA, the remainder of the TRIzol/chloroform mixture was used for DNA isolation following the Invitrogen protocol. For each condition, *n* = 3 DNA samples were obtained. The DNA was precipitated with 100% ethanol, and then centrifuged and washed twice with 0.1 M sodium citrate (Sigma) in 10% ethanol, pH 8.5, and once with 75% ethanol. After resuspension in 8 mM NaOH, and pH adjustment to 7.5–8.5 with HEPES (ThermoFisher), DNA purity and concentration was measured with Nanodrop. DNA was then digested with S1 nuclease (ThermoFisher) and alkaline phosphatase (Sigma). The 8-OHdG ELISA kit (Abcam) was performed following the manufacturer’s instructions. 8-OHdG concentration was measured at 450 nm (A_450_) dependent on the enzymatic color reaction of 3,3′,5,5′-tetramethylbenzidine (TMB) Substrate on a SpectraMax M5 UV-Vis Spectrophotometer (Molecular Devices).

### Statistics

For OWH LDH % cytotoxicity analyses with *n* = 18 samples, because the NT control failed to pass the normality test, we performed statistical analyses with the Kruskal-Wallis test with Dunn’s test for multiple comparisons. For LDH *n* = 18 samples, data were plotted as mean ± standard error of the mean (SEM) error bars displayed on the graphs, unless the error bars were too small to visualize. For the HC vs OWH LDH % cytotoxicity, RT-PCR, and 8-OHdG comparisons, we assumed normality and assessed significance using parametric unpaired t-tests with Welch’s correction. For samples assessed with t-tests, data was plotted as mean ± SEM error bars displayed on the graphs, unless the error bars were too small to visualize. We reported statistical significance at two *p*-value levels: *p* < 0.05 (*) and *p* < 0.001 (**).

## Results

### Establishment of the MSG-induced excitotoxicity slice model

**Fig. 1:**
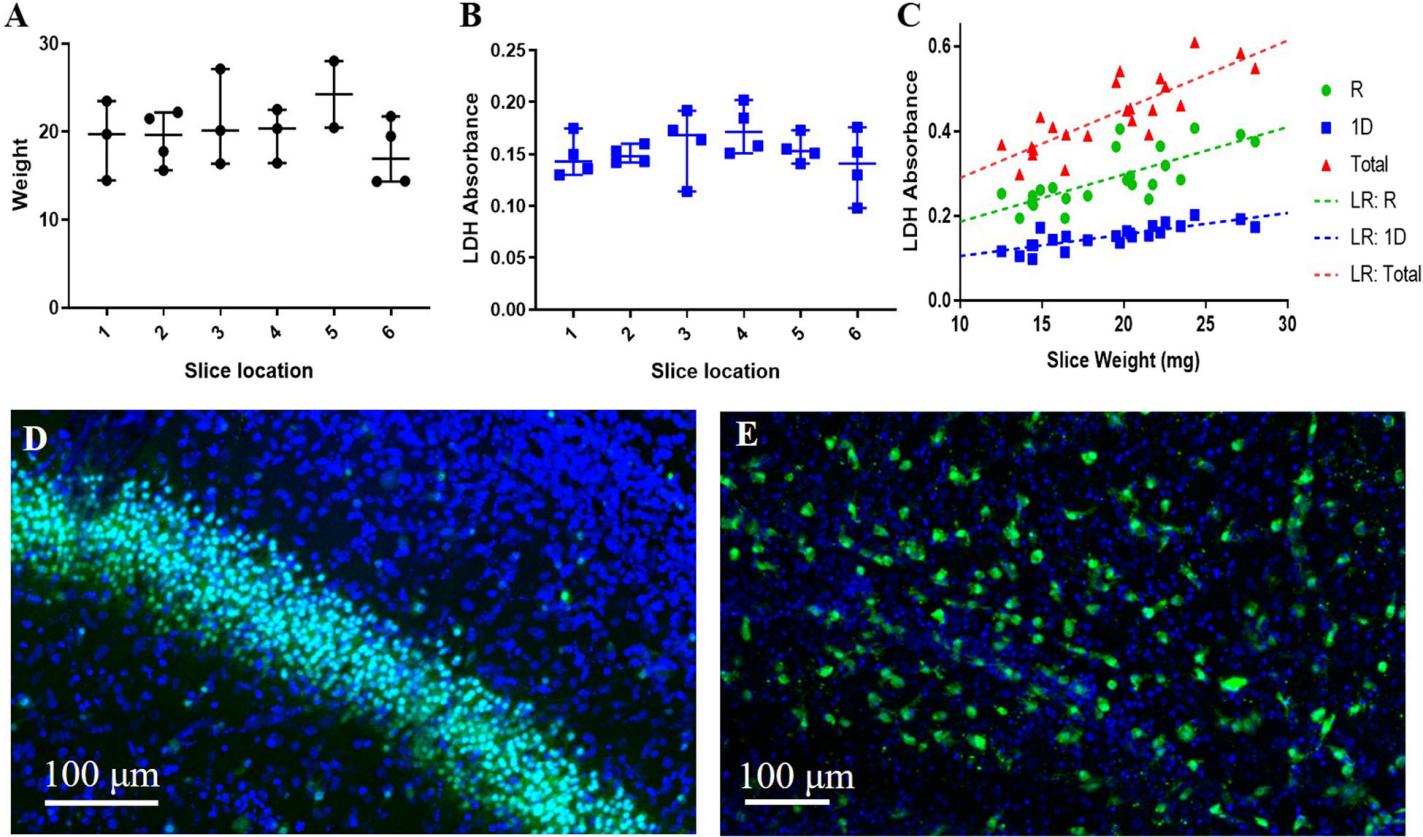
Establishment of the ex vivo whole hemisphere slice model. **a** Brain hemisphere tissue weight based on slice location (*n* = 2–4, median with 95% CI). **b** LDH absorbance variability based on slice location (*n* = 4, median with 95% CI). **c** LDH absorbance of slices as a function of slice weight. R: overnight rest; 1D: after 24 h; LR: linear regression. Cellular morphology of **d** neurons stained with NeuN (green) and **e** microglia stained with Iba + (green) after 6 h culturing. Slices were additionally stained with the cell nuclei marker, DAPI (blue). Scale bar is 100 μm

While OHC and cortical slices are widely used in the field, OWH slices have not previously been used for studying neurological disease [18]. Therefore, we determined the variation of OWH slice weight to support OWH reliability for quantitative analyses. Slice weight and LDH release after 1 day of culturing exhibited no significant difference based on slice location, as determined by the Kruskal-Wallace with Dunn’s Multiple Comparisons test (Fig. 1a and b respectively; *p* > 0.9999 for all). Slices were numbered 1 through 6 based on rostral to caudal slice location, starting with the first slice to contain a full hippocampal section. Slices exhibited a linear correlation in LDH release during the overnight rest period (*r*^2^ = 0.5465), after 1D of culturing (*r*^2^ = 0.6416), and during the combined rest +1D Total (*r*^2^ = 0.6733) as a function of slice weight (Fig. 1c). After the slice preparation process of slicing, resting overnight, and culturing for 6 h, non-treated slices exhibited healthy cellular morphology, as indicated by morphologically normal NeuN+ neurons and Iba-1+ microglial cells. Representative images from the hippocampus are shown (Fig. 1d-e).

**Fig. 2:**
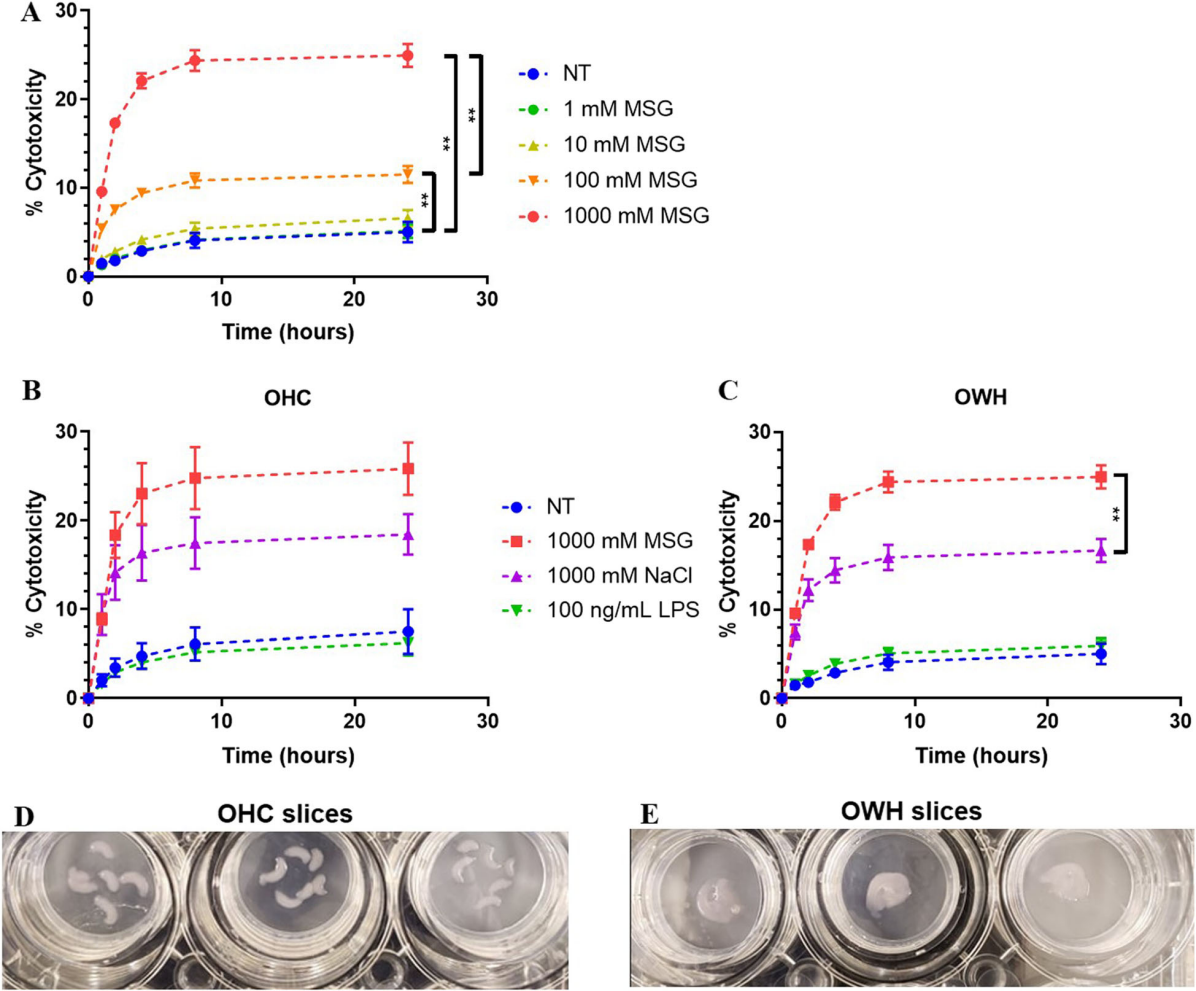
Establishment of the MSG-induced excitotoxicity slice model. Percent cytotoxicity was calculated from LDH absorbance for **a** slices treated with gradient of MSG concentrations (*n* = 18; mean ± SEM), and **b** organotypic hippocampal (OHC) (*n* = 3; mean ± SEM) and **c** organotypic whole hemisphere (OWH) (*n* = 6 NaCl, LPS; *n* = 18 NT, MSG; mean ± SEM) ex vivo slice cultures treated with various exposures, referenced to the 24 h cumulative 1% TX LDH absorbance as 100%. Representative photos of **d** OHC slice cultures and **e** OWH slice cultures

Slices were incubated with a gradient of MSG concentrations to induce excitotoxicity (Fig. 1a). 100 mM and 1000 mM MSG exhibited a significant increase in cell death compared to the NT control (*p* < 0.0001 for both). Because 1000 mM MSG exhibited a greater increase in cytotoxicity than 100 mM MSG exhibited (*p* < 0.0001; parametric unpaired t-test with Welch’s correction), 1000 mM MSG was used as the concentration for LDH cytotoxicity studies, with all non-specified MSG conditions at 1000 mM. NT slices exhibited 5.04% cytotoxicity compared to TX-induced complete cell death (Fig. 1a). Because OHC slices are the most commonly used organotypic brain slice platform in the field, OWH slice responses to exposures were compared to OHC slice responses to establish OWH slices as a reliable and alternative model [18]. Compared to the respective condition of 1000 mM MSG, 1000 mM NaCl, and 100 ng/mL LPS, OHC and OWH slices exhibited no significant difference in cytotoxicity profiles. 1000 mM MSG-treated slices elicited 8.28% greater cytotoxicity than 1000 mM NaCl-treated slices (*p* = 0.0004). Figure 2d and e display representative photos of OHC slice cultures and OWH slice cultures, respectively.

**Fig. 3:**
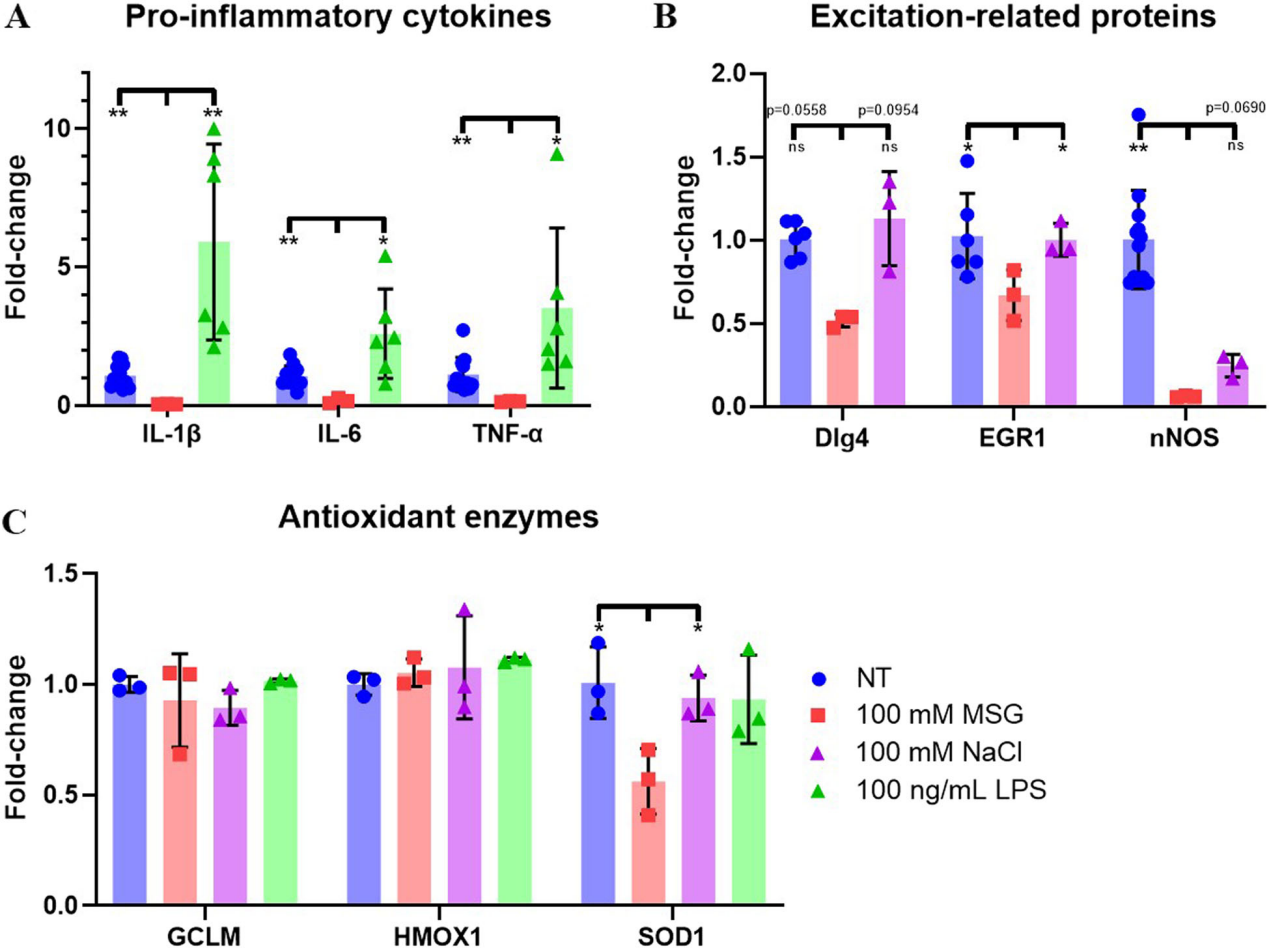
Fold-changes of mRNA markers for inflammation-, excitation-, and antioxidant-related proteins of NT, 100 mM MSG, 100 mM NaCl, and 100 ng/mL LPS slices at 6 h. **a** Fold-change of mRNAs for inflammatory cytokines of NT, MSG, and LPS slices (*n* = 12 NT; *n* = 3 MSG; *n* = 6 LPS; mean ± SEM). **b** Fold-change of mRNAs for excitation-related proteins of NT, MSG, and NaCl slices (*n* = 6–12 NT; *n* = 3 MSG and NaCl; mean ± SEM). **c** Fold-change of mRNAs for antioxidant enzymes of NT, MSG, NaCl, and LPS slices (*n* = 6 NT; *n* = 3 MSG, NaCl, and LPS; mean ± SEM). ns: not significant

For RT-PCR analyses, 100 mM MSG and NaCl were used instead of 1000 mM due to the fact that at 1000 mM Na^+^ concentrations, mRNA expression for all antioxidant enzymes was almost completely suppressed (Additional file 1: Figure S3). After 3 h incubation in NT, 100 mM MSG, 100 mM NaCl (hyperosmolar stress positive control), or 100 ng/mL LPS (inflammation positive control) conditions, and then 6 h after exposure removal, slices were collected for RT-PCR analysis. MSG-treated slices exhibited a significant decrease in pro-inflammatory cytokine mRNA expression in comparison to NT and LPS-treated slices for IL-1β (NT: *p* < 0.0001; LPS: *p* = 0.0097), IL-6 (NT: *p* < 0.0001; LPS: *p* = 0.0142), and TNF-α (NT: *p* = 0.002; LPS: *p* = 0.0354) (Fig. 3a). Furthermore, MSG-treated slices exhibited a fold-decrease in expression in comparison to NT slices for the excitation-related mRNAs EGR1 (*p* = 0.0122) and nNOS (*p* < 0.0001), and to NaCl-treated slices for EGR1 (*p* = 0.0112) (Fig. 3b). In comparison to NT slices, there were no significant differences in expression of antioxidant enzymes GCLM and HMOX1 for any treatment condition. MSG exposure elicited a significant decrease in SOD1 expression compared to NT (*p* = 0.0117) and NaCl-treated slices (*p* = 0.0271). 100 mM NaCl-exposed and 100 μM NMDA-exposed slices also exhibited a significant fold-decrease in pro-inflammatory mRNA expression (Additional file 1: Figure S1). 100 μM NMDA-exposed slices showed no change in Dlg4, EGR1, or antioxidant enzyme mRNA expression, and a decrease in nNOS mRNA expression (Additional file 1: Figure S2).

### Superoxide dismutase antioxidant effects on MSG-induced excitotoxicity

**Fig. 4:**
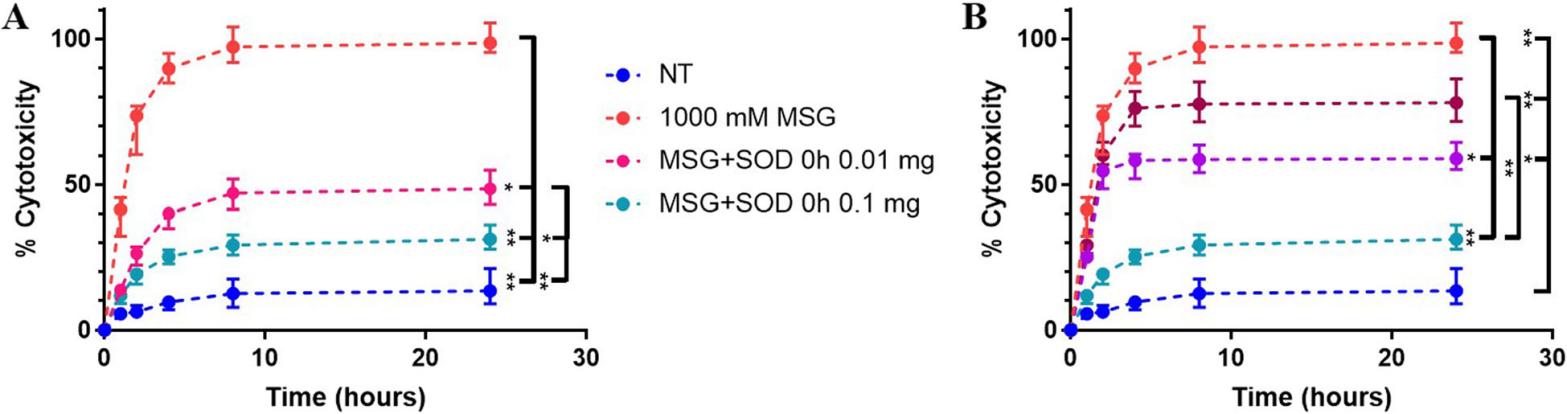
Therapeutic effect of SOD application on MSG-induced excitotoxic OWH slices. Percent cytotoxicity from LDH release was calculated with 24 h cumulative LDH release from 1000 mM MSG-induced slices as 100%. **a** MSG-induced slices treated with 0.01 mg and 0.1 mg SOD, and **b** MSG-induced slices treated with 0.1 mg SOD at 0 h, 2 h, and 4 h timepoints (*n* = 18; median with 95% CI)

Application of SOD to MSG-induced excitotoxic OWH slices reduced cell death. Application of 0.01 mg and 0.1 mg SOD at 0 h reduced toxicity to 43.72% (*p* = 0.0304) and 23.99% (*p* < 0.0001) respectively (Fig. 4a), relative to 1000 mM MSG LDH release as 100% cytotoxicity. There was no significant difference between the NT (18.02%) and MSG + 0.1 mg SOD sample (*p* = 0.5858). SOD’s therapeutic effect was further explored based on timing of administration. Whether applied at 0 h, 2 h, or 4 h after exposure to MSG, cytotoxicity was reduced compared to MSG without SOD treatment and reached a plateau by 24 h (Fig. 4b). Administration of SOD at 2 h significantly decreased cytotoxicity to 59.39% (*p* = 0.0005), while 4 h treatment reduced toxicity to 79.24%, but not significantly (*p* = 0.5092).

**Fig. 5:**
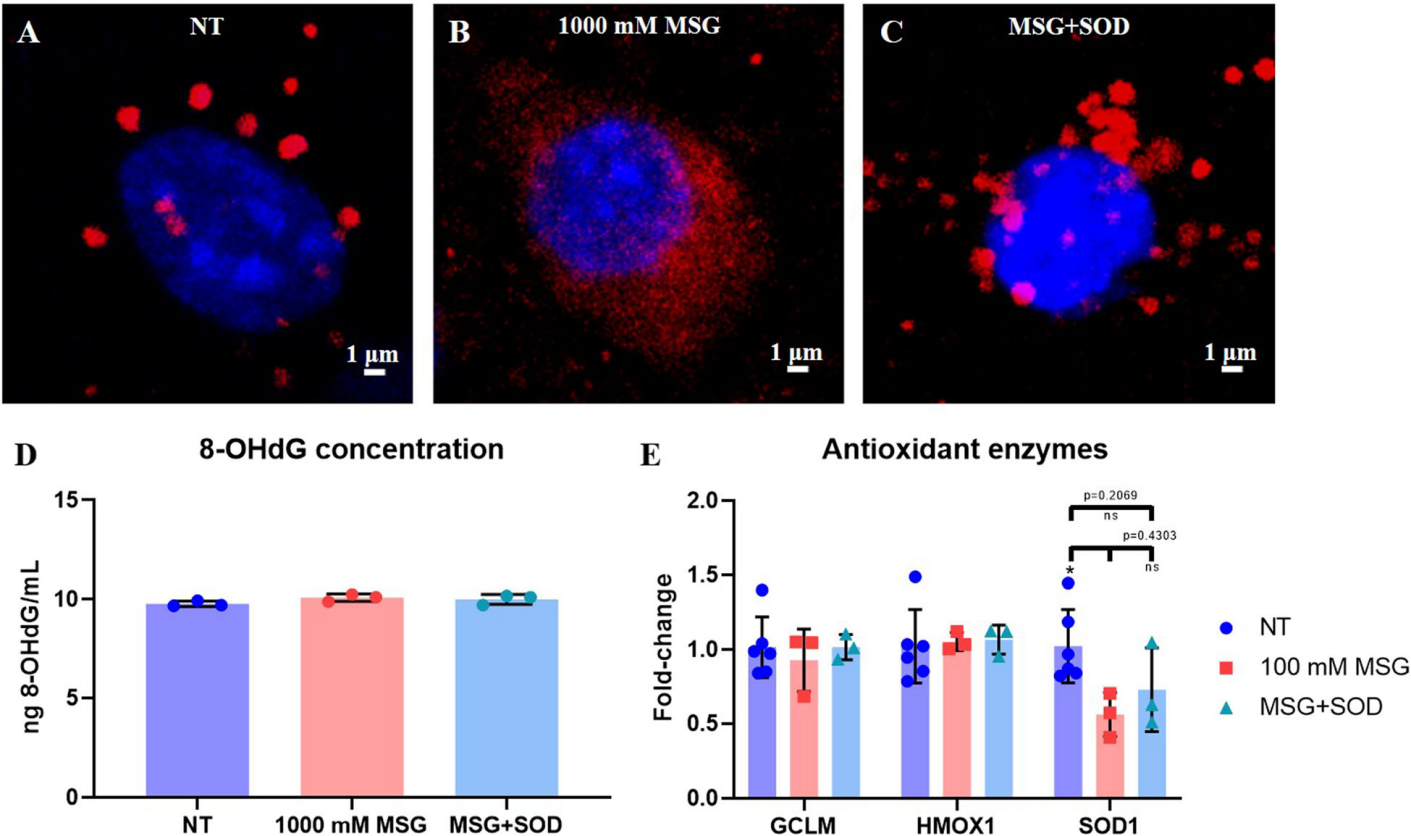
The exploration of mechanistic effects of SOD administration. Mitochondrial morphology of **a** NT, **b** 1000 mM MSG, and **c** 1000 mM MSG with 0.1 mg SOD applied at t = 0 h slices. Slices were stained with MitoTracker Deep Red FM (red) and DAPI (blue). Scale bar is 1 μm. **d** Concentration of 8-OHdG in DNA extracted from NT, 100 mM MSG, and 100 mM MSG + 0.1 mg SOD/slice conditions at 6 h as determined by 8-OHdG ELISA kit (*n* = 3; mean ± SEM). **e** Antioxidant enzyme mRNA fold-change expression at 6 h for NT, 100 mM MSG, and 100 mM MSG + 0.1 mg SOD/slice (*n* = 3–6; mean ± SEM)

Furthermore, SOD treatment improved mitochondrial health. Figure 5a-c display representative images of a cell from non-treated, 1000 mM MSG-treated, and 1000 mM MSG + 0.1 mg SOD-treated slices, where cell nuclei are stained with DAPI and mitochondria with MitoTracker Deep Red FM. Compared to control slices, MSG-treated slices exhibited extensive fragmentation of mitochondria, yielding more numerous and smaller mitochondria. Upon application of 0.1 mg SOD at t = 0 h to the slice incubated with MSG from -3 h to 0 h, a return to non-treated mitochondrial morphology was observed, with larger and less numerous mitochondria in MSG + SOD-treated slices than the observed mitochondria incubated with MSG alone. To investigate the role of peroxynitrite-mediated toxic downstream products, 8-OHdG concentration in DNA extracted from NT, MSG, and MSG + SOD slices at the 6 h timepoint was assessed. However, there was no significant difference in any of the three conditions, with concentrations of 9.770, 10.080, and 9.995 ng/mL respectively (Fig. 5d). MSG did not elicit a significant increase in 8-OHdG concentration compared to NT slices (*p* = 0.0895), and SOD did not significantly decrease 8-OHdG concentration compared to MSG alone (*p* = 0.6767). Treatment of MSG slices with SOD did not significantly affect the mRNA expression of SOD1 compared to MSG (*p* = 0.4303) (Fig. 5e).

## Discussion

Glutamate excitotoxicity is a factor in the etiology of multiple neurological disorders, with multiple proposed pathways involved in inflicting damage. Here, we use an OWH brain slice model as a high-throughput tool for monosodium glutamate (MSG)-induced excitotoxicity disease model development and therapeutic efficacy screening. The ex vivo model has enabled us to isolate components of the naturally convoluted disease processes of glutamate excitation, inflammation, and hyperosmolar stress to better understand and treat excitotoxicity. Extracellularly administered SOD was effective in inhibiting cell death and restoring healthy mitochondrial morphology after exposure to MSG. However, based on early 8-OHdG concentrations, we also show that peroxynitrite-mediated DNA damage may not play a primary role in initiating excitotoxic cell death in this model.

Organotypic ex vivo slices model the multi-cell type and 3D architecture interactions of in vivo systems that in vitro immortalized cell lines or primary cell culture cannot, and can be used to effectively screen therapeutic strategies before evaluating efficacy in vivo [18]. Organotypic brain slice models enable more representative study of excitotoxicity, where glutamate and oxidative stress regulation involves multiple cell types [20, 21]. Even with improving technologies permitting in vitro co-cultures, culturing neurons, microglia, astrocytes, oligodendrocytes, pericytes, and brain microvascular endothelial cells together is still not feasible [22, 23]. Organotypic brain slices have commonly been dissected to culture the hippocampus, cortex, or striatum in isolation to study cell population subsets using single-cell resolution methods such as electrophysiology or IF [24, 25]. However, whole hemisphere cultures have not been popularized, missing the opportunity for performing bulk and regionally-variable quantitative analyses. OWH slice culturing also utilizes a greater quantity of processable tissue from an individual brain than OHC slice culturing, reducing animal numbers needed for multi-modal tissue processing such as LDH, RT-PCR, and ELISA. Regardless of rostral to caudal position, the six coronal OWH slices containing the hippocampus used in our studies exhibit minimal variation in tissue weight, providing consistency for quantitative analyses (Fig. 1). One P14 brain hemisphere can produce about six OWH slices containing the hippocampus before the enlargement of ventricles affects slice separation integrity. However, one OWH slice occupies approximately the same area as six OHC slices, requiring one whole hemisphere to obtain a comparable volume of hippocampal tissue (Fig. 2c-d). Furthermore, OWH slices behave similarly in cytotoxicity response to MSG, NaCl, and LPS compared to OHC slices (Fig. 2b-c).

We applied 1–1000 mM MSG to induce glutamate excitotoxicity (Fig. 2a). Although presynaptic neuronal vesicles contain glutamate concentrations of ~ 70–200 mM [26], we selected 1000 mM MSG dose for LDH studies to examine a near-maximal cytotoxic response to MSG for therapeutic analyses. Additionally, we analyzed mRNA expression levels of slices after exposure to 100 mM MSG, 100 mM NaCl, 100 ng/mL LPS, or 100 μM NMDA. NaCl and NMDA exposures were performed to discern the effects of glutamate receptor activation as opposed to hyperosmolar sodium stress alone, while LPS treatment was performed to compare MSG excitotoxicity to a model of neuroinflammation [27, 28]. At 1000 mM salt concentrations the housekeeping gene GAPDH retained baseline expression levels, suggesting that mRNA transcript stability was maintained; however, expression of the target transcripts under investigation was reduced compared to NT slices for both MSG and NaCl treatment, preventing meaningful differentiation between the two exposures due to excitotoxicity mechanisms (Additional file 1: Figure S3) [29, 30]. It is important to emphasize that all RT-PCR work was conducted with only female brain slices, and that there are significant gender differences in vulnerability to excitotoxicity, oxidative stress resistance, and mitochondrial function [31–34]. MSG-induced mRNA responses in males could vary greatly from the results obtained in females, and hence are not encompassed in this study, but need to be further explored.

In vivo, excitotoxicity and inflammation are intimately intertwined where excitotoxicity can cause or be caused by inflammation, and both involve oxidative stress [35–38]. After excitotoxic neuronal death, cells release pro-inflammatory cytokines and danger-associated molecular patterns that activate microglia and astrocytes [39–41]. Activated glia proliferate and migrate to damaged areas, and release molecules that exacerbate excitotoxicity, including NOX2-generated O_2_^−^, excitatory glutamate, and TNF-α [42–47]. These interrelated pathologies make identification of the primary pathological players in excitotoxicity a complex process. By administering exogenous MSG, we induced excitotoxicity without the induction of pro-inflammatory cytokines. In our work, while LPS elicited upregulation of inflammatory IL-1β, IL-6, and TNF-α, MSG treatment significantly decreased expression for these same cytokines (Fig. 3a). A similar decrease in pro-inflammatory cytokine expression was observed with exposure to 100 mM NaCl and 100 μM NMDA (Additional file 1: Figure S1). The lack of inflammatory cytokine expression suggests glial NOX2-generated O_2_^−^ may not be a requisite contributor to pathological O_2_^−^ levels in excitotoxic death. The OWH model can therefore assist in isolating the primary excitotoxic core injury from the inflammatory penumbra observed in vivo [48].

One potential confounder of the MSG model is the associated sodium load. Although glutamic acid is found physiologically in its anionic form, exogenous sodium is also a relevant aspect of the model, as extensive sodium uptake leading to cell swelling and lysis is an important phenomenon in necrotic excitotoxic death [6]. Additionally, hyperosmolarity and excitotoxicity converge in pathological mechanisms through dysregulated calcium homeostasis and mitochondrial oxidative stress [49]. Morland et al. have shown that the replacement of chloride with the inert anion gluconate had no effect on sodium hyperosmolar neurotoxicity [50]. Therefore, comparison of MSG versus NaCl allows us to observe the effects of specifically the glutamate anion, where MSG exposure elicited greater cytotoxicity than NaCl exposure (Fig. 2c). Hyperosmolar conditions result in the uptake of anionic osmolytes including chloride, glutamate, and taurine, to minimize water efflux and cell shrinkage [50]. At 0 h, upon removal of 1000 mM MSG or NaCl and return to normo-osmolar conditions, cells then efflux accumualted osmolytes including glutamate, leading to greater extracellular glutamate levels conducive to further excitotoxicity. It reasonably follows that the presence of excess glutamate in MSG-treated slices could lead to exacerbated uptake and subsequent efflux of glutamate back into the extracellular space for increased toxicity.

Upon oxidative stress due to injury or inflammation, the antioxidant response element known as nuclear factor erythroid 2-related factor 2 (NRF2) translocates to the nucleus to upregulate the transcription of HMOX1 and GCLM antioxidants [51]. The OWH slices are already in a state of acute oxidative stress due to injury in the tissue chopping process, which overnight resting partially mitigates [27]. HMOX1 and GCLM expression in MSG slices exhibited no significant difference from the NT condition (Fig. 3c). Under oxidative stress conditions, many transcriptional pathways downregulate to prioritize only survival-related proteins [51, 52]. The retention of NRF2 target expression, but not pro-inflammatory mRNA expression, suggests that MSG mediates damage primarily through oxidative stress independent of inflammation. However, there was a decrease in SOD1 mRNA expression for 100 mM MSG-treated slices compared to NT and 100 mM NaCl-treated slices (Fig. 3c). The severe MSG-induced oxidative stress could upregulate activating factor 1 (AP-1) and AP-2, repressing SOD1 mRNA expression [9, 53, 54].

The 100 mM MSG slices reduced expression of excitation-related transcripts, decreasing EGR1 mRNA expression compared to NT and NaCl-treated slices, and decreasing nNOS expression compared to NT slices (Fig. 3b). EGR1 is an immediate early gene (IEG) indicator of excitation activity, and Dlg4 is a NMDAR-associated synaptic scaffolding protein that recruits nNOS to produce nitric oxide (NO) [55, 56]. OWH MSG exposure potentially preferentially damaged neurons compared to other cell populations [57]. The MSG-induced oxidative stress could interfere with neuronal RNA stability and transcription [29, 58–61], or selectively increase neuronal death, ultimately reducing the neuronal population contributions to mRNA transcripts of excitation-related proteins. However, in vivo studies show increases in expression of nNOS and other IEGs after excitotoxic insults [62–65]. The discrepancy could be explained by the direct exposure of OWH slices to MSG throughout the entire slice, as opposed to exposure only at the localized primary insult in vivo. The primary insult elicits increased excitatory activity among neighboring neurons, and hence could explain overall nNOS and IEG mRNA upregulation in vivo [62]. The definitive cause of reduced mRNA expression of excitation-related proteins in MSG-exposed OWH slices requires further exploration.

Despite the severe oxidative stress environment and decreased SOD1 mRNA expression due to MSG exposure, we found that exogenously-applied SOD provided a neuroprotective effect against MSG-induced excitotoxicity. Furthermore, the decrease in cytotoxicity was directly dependent on the timing of SOD administration and elicited a plateau in cytotoxicity whether administered at 0 h, 2 h, or 4 h (Fig. 4a-b). This further confirms the efficacy of SOD against excitotoxicity, supporting previous in vitro and in vivo studies of SOD amelioration of excitotoxic death [15, 16, 66]. Although administered extracellularly, SOD effectively inhibits O_2_^−^ toxicity, suggesting that extracellular delivery is sufficient for inhibition, as opposed to requiring targeted cellular uptake and mitochondrial localization [67]. For potential clinical translation of SOD for neurological disorders, it is important to note that macromolecular enzymes are prone to protease degradation and fail to cross the blood-brain barrier. Therefore, SOD would require assistance in drug delivery to reach the disease site through the use of nanoparticles or alternative approaches, before its exogenous therapeutic effect in brain tissue could be realized [68, 69]. The therapeutic effect of SOD on MSG-induced excitotoxicity was further confirmed by observing mitochondrial morphology (Fig. 5a-c). It is important to note that not all NT and MSG + SOD mitochondria were non-fragmented, and not all MSG mitochondria were fragmented, but Fig. 5a-c represents the large majority of cells imaged. During oxidative stress, mitochondria undergo extensive fission to isolate damaged portions of the mitochondria, resulting in more numerous and smaller mitochondria as seen in MSG-treated slices (Fig. 5b) [70]. By scavenging O_2_^−^, SOD reduces mitochondrial damage, reducing extensive fission to yield mitochondria resembling those of NT cells (Fig. 5c). Interestingly, the SOD reaction produces 50% molar concentrations of H_2_O_2_, which does not appear to affect overall cell viability in our model.

Studies have shown that elevated O_2_^−^ production from mitochondrial respiration in combination with elevated NO production generates the highly reactive species peroxynitrite (ONOO^−^) [71]. Peroxynitrite reacts with biological molecules causing protein, lipid, and nucleic acid oxidative and nitrosative damage [71]. However, we observed no significant changes in 8-OHdG, a ONOO^−^-mutated DNA product, concentration at 6 h (Fig. 5d). This suggests that ONOO^−^ may not hold a primary role in the early pathogenesis of excitotoxicity. Supporting this idea, Choi et al. demonstrated that nNOS and NOX2 inhibition prevented NMDAR hypersensitivity but ONOO^−^ inhibition did not, suggesting ONOO^−^ is downstream of the cause of excitatory dysfunction [72]. The absence of a positive control for 3-NT downstream products is a limitation of our analysis. Future work could confirm the lack of a role for peroxynitrite in initial excitotoxic injury with a model that differentially expresses 8-OHdG at later timepoints beyond 6 h. Despite SOD providing a neuroprotective effect in reducing cell toxicity, SOD did not restore SOD1 mRNA expression. However, SOD1 expression was no longer significantly different from NT slices for SOD-treated slices (Fig. 5e). We suspect SOD interrupts the pathological pathway towards cell death, rather than inhibiting initial excitation pathways when applied 3 h after MSG treatment. It remains possible that earlier SOD administration could more effectively inhibit MSG pathology and SOD1 mRNA downregulation. Whether exogenous addition of SOD revitalizes native antioxidant systems requires further exploration.

## Conclusion

Using OWH brain slice models, we can bypass the many obstacles associated with drug delivery to the brain to reliably screen therapeutics in high-throughput fashion prior to in vivo evaluation. OWH brain slices exhibit healthy cellular architecture after culture and respond to exposures similarly to widely studied OHC slices, while reducing animal numbers for quantitative analyses. With RT-PCR, distinguishing glutamate excitotoxicity from inflammation and sodium hyperosmolarity enables us to better understand primary pathological mechanisms in excitotoxicity. SOD administration reduced cytotoxicity and restored healthy mitochondrial morphology. Supported by the absence of increased 8-OHdG residues upon MSG treatment, peroxynitrite-mediated damage may not play a primary role in initial excitotoxic damage. SOD can help inhibit cell death, but a fully effective therapeutic strategy could benefit by utilizing a combinatorial therapy with another drug that targets excessive neuroinflammation, and delivery within a nanoparticle platform. Altogether, SOD is a very promising enzyme therapeutic for combating excitotoxicity in a plethora of neurological diseases.

## Supplementary information

**Additional file 1: Figure S1.** Fold-changes of pro-inflammatory mRNAs for NT, 100 μM NMDA, and 100 mM NaCl slices at 6 h (*n* = 3–12). **Figure S2.** Fold-changes of excitation-related proteins and antioxidant enzyme mRNAs mRNAs for NT and 100 μM NMDA slices at 6 h (*n* = 3–12). **Figure S3.** Fold-changes of antioxidant mRNAs for NT, 1000 mM MSG, and 1000 mM NaCl slices at 6 h (*n* = 1).

## Data Availability

The datasets used and/or analyzed during the current study are available from the corresponding author on reasonable request.
