# Supplementary material for: Superoxide dismutase reduces monosodium glutamate-induced injury in an organotypic whole hemisphere brain slice model of excitotoxicity
Source: J Biol Eng. 2020 Feb 4;14:3. doi: 10.1186/s13036-020-0226-8 (PMC7001228; doi:10.1186/s13036-020-0226-8)
Supplement: Supplementary file 1 — Additional file 1: Figure S1. Fold-changes of pro-inflammatory mRNAs for NT, 100 μM NMDA, and 100 mM NaCl slices at 6 h (n = 3–12). Figure S2. Fold-changes of excitation-related proteins and antioxidant enzyme mRNAs mRNAs for NT and 100 μM NMDA slices at 6 h (n = 3–12). Figure S3. Fold-changes of antioxidant mRNAs for NT, 1000 mM MSG, and 1000 mM NaCl slices at 6 h (n = 1). [file 13036_2020_226_MOESM1_ESM.docx]

**SUPPLEMENTARY FIGURES**

**
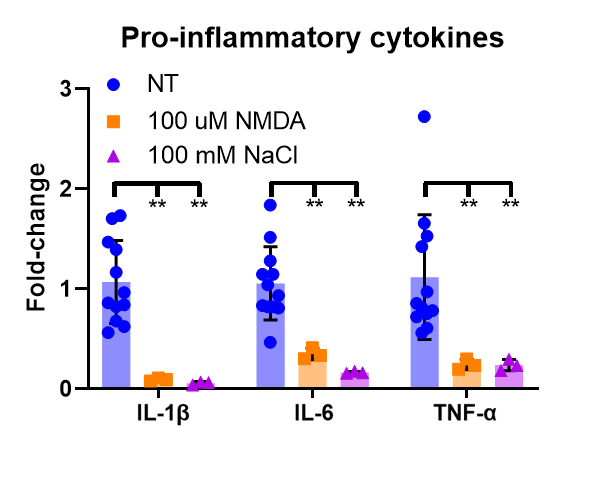
**

*Figure S1:* Fold-changes of pro-inflammatory mRNAs for NT, 100 μM NMDA, and 100 mM NaCl slices at 6h (n=3-12).

**
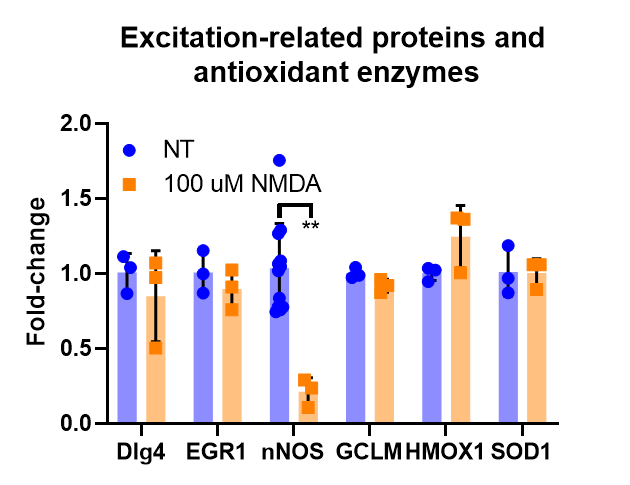
**

*Figure S2:* Fold-changes of excitation-related proteins and antioxidant enzyme mRNAs mRNAs for NT and 100 μM NMDA slices at 6h (n=3-12).

*
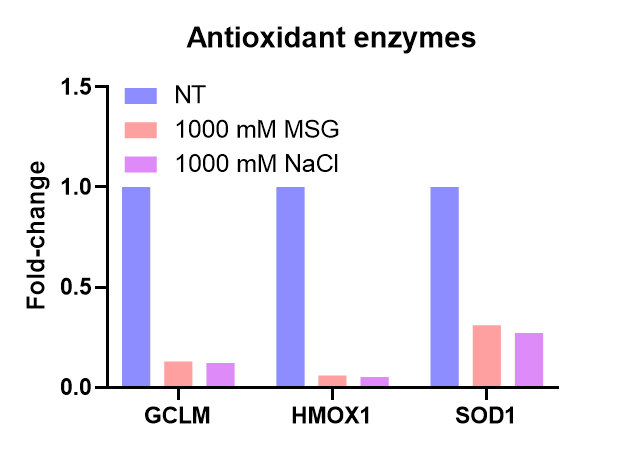
*

*Figure S3:* Fold-changes of antioxidant mRNAs for NT, 1000 mM MSG, and 1000 mM NaCl slices at 6h (n=1).
